# Prenatal and early-life exposure to traffic-related air pollution and allergic rhinitis in children: A systematic literature review

**DOI:** 10.1371/journal.pone.0284625

**Published:** 2023-04-20

**Authors:** Lifang Liu, Jingxuan Ma, Shanshan Peng, Linshen Xie

**Affiliations:** West China School of Public Health and West China Fourth Hospital, Sichuan University, Chengdu, China; Kyung Hee University School of Medicine, REPUBLIC OF KOREA

## Abstract

**Background:**

Traffic-related air pollution (TRAP) is hypothesised to play a role in the development of allergic rhinitis (AR). Prenatal and early-life exposure to traffic-related air pollution is considered critical for later respiratory health. However, we could not find any articles systematically reviewing the risk of prenatal and early-life exposure to traffic-related air pollution for allergic rhinitis in children.

**Methods:**

A systematic literature search of PubMed, Web of Science and Medline was conducted to identify studies focused on the association between prenatal and early-life exposure to TRAP and AR in children. Other inclusion criteria were: 1) original articles; 2) based upon prospective or retrospective studies or case-control studies; and 3) publications were restricted to English. Literature quality assessment was processed using the Newcastle-Ottawa Scale (NOS) evaluation scale. This systematic literature review has been registered on the prospero (crd.york.ac.uk/prospero) with the following registry number: CRD42022361179.

**Results:**

Only eight studies met the inclusion criteria. The exposure assessment indicators included PM_2.5_, PM_2.5_ absorbance, PM_10_, NO_x_, CO, and black carbon. On the whole, exposure to TRAP during pregnancy and the first year of life were positively associated with the development of AR in children.

**Conclusions:**

This systematic review presents supportive evidence about prenatal and early-life exposure to TRAP and the risk of AR in children.

## Introduction

Allergic rhinitis (AR) is defined as a chronic, waxing/waning, immunoglobulin E (IgE) -based inflammation in the nasal mucosa. It occurs in response to typically innocuous environmental proteins such as mites, pollen, and animal skin dander and has a variety of manifestations including swollen nasal mucosa, nasal hypersecretion, rhinorrhea, sneezing, and itching [1]. Among childhood allergic diseases, allergic rhinitis has become the most common manifestation and a major health problem. In recent years, the prevalence of childhood allergic rhinitis is about 39.7% worldwide [2]. The prevalence of allergic diseases in children and adolescents continues to increase, particularly in low and middle-income countries globally. Allergic rhinitis seriously impacts the growth of children by causing various problems, such as sleep disturbance, emotional stress, and impairment of school productivity [3], also increases the incidence of bronchial asthma, nasal polyps, allergic conjunctivitis and the risk of sinus and middle ear infections [1]. Changes in environmental factors are considered to be the most significant cause of the increase and this contribution might be partly attributed to increased traffic-related air pollution (TRAP) [4].

There is an emerging body of evidence that chronic exposure to air pollution, especially TRAP, can lead to allergic diseases [5–10]. However, the limited evidence for the association between long-term exposure to air pollution and AR is equivocal. TRAP is a complex mixture of gases and suspended particulate matter (PM) produced by motor vehicles through combustion and non-combustion [11]. There is a wide range of TRAP, the main ones being nitrogen oxides (NO_x_), especially nitrogen dioxide (NO_2_), elemental carbon (EC), ultrafine particles (UFP), fine particle matter (PM_2.5_), coarse particle matter (PM_10_) and carbon dioxide (CO_2_). These are considered to be primary pollutants that can be emitted directly as tailpipe emissions, with non-tailpipe emissions arising mainly from the resuspension of dust, wear and tear of vehicle parts and road surfaces. The contribution of TRAP to air pollution has reached a high proportion in some major cities worldwide and has become a major source of air pollution in cities [12].

Since the emission from traffic sources is located at high levels near the human respiratory zone, it is closely related to public health. In light of the process of lung development begins in early embryonic life and continues through adolescence, exposure to environmental hazards during any of the phases of development may result in altered developmental programming which is responsible for increased risk of diseases in later life [13]. Environmental challenges during pregnancy and the early life period have long been thought to modulate susceptibility to some chronic diseases in later life, which is commonly referred to as the “Barker hypothesis” or “developmental plasticity” [14]. Prenatal and early-life are critical periods for lung morphogenesis and maturation, during which exposure to environmental pollutants may lead to structural alterations and altered repair mechanisms that are long-term functional changes of organs, resulting in long-lasting impairment of resistance to infection and increases the risk of allergies later in life [15–17]. Several epidemiological studies have reported that exposure to TRAP during pregnancy and early-life increases the risk of allergy to multiple allergens [18]. When exposure occurs in late childhood or adolescence, the effects on lung function are less severe and the condition is reversible in the absence of continued stimuli, suggesting that exposure in late childhood or adolescence may not lead to long-term respiratory deficiencies [13, 19]. Therefore, we reasonably conjecture that prenatal and early-life exposure to TRAP seems to be of greater significance than later exposure.

Few studies have addressed AR as an endpoint. More recently, exposure to fine particle matter (PM_2.5_), black carbon (BC), nitrogen dioxide (NO_2_) during pregnancy and the first 3 years of life was found to be associated with increased morbidity of AR [2, 8, 20–22]. A positive association between PM_2.5_ absorbance and the incidence of AR was also described [23].

The effects of prenatal and early-life exposure to TRAP and allergic diseases (including asthma and atopic eczema) have been largely established [7, 15–17, 24–26], while a gap in evidence on allergic rhinitis exists. Therefore, we conducted a systematic review to report the findings of existing studies and further clarify the relationship between TRAP and AR.

## Material and methods

This systematic literature review complies with the PRISMA 2020 guidelines [27]. The protocol has been registered on the prospero platform(crd.york.ac.uk/prospero) with the following registry number: CRD42022361179.

### Search strategy and search terms

We systematically searched PubMed, Web of Science and Medline (from March 2000 to September 2022) to identify studies on the association between AR and TRAP during pregnancy and early life. The original search formula was composed of the following keyword combinations: TRAP terms (“PM_2.5_”, “NO_x_”, “NO_2_”, “traffic-related air pollution”, “traffic pollutant*”, “vehicle emission”, “nitrogen oxides”, “particulate matter”, “traffic exposure”, “automobile emission”, “traffic emission”, “proximity to roadways”, “proximity to major roads”, “ambient air pollution”) and allergic rhinitis terms(“allergic rhinitis”, “rhinitis, allergic”) and participants terms (“child health”, “childhood”, “adolescents”, “teenagers”, “children”, “pre-school children”, “prenatal exposure*”, “early life exposure*”, “pregnancy”, “preconceptional exposure*” and “pregnancy exposure*”). In addition, we conducted a manual search of the references to original studies and reviews that fit the topic to ensure that no eligible studies were missed.

### Study selection and inclusion/exclusion criteria

Study selection was conducted by two reviewers (Lifang Liu and Jingxuan Ma) independently. Any disagreements were discussed and resolved with full reference to opinion of the third reviewer (Shanshan Peng). Eligibility was assessed on the basis of the title or abstract and, if necessary, the full text. Articles were eligible for this review if they identified an association between AR and air pollution during pregnancy and early-life in the title or abstract. Other inclusion criteria were: 1) original articles; 2) based upon prospective or retrospective studies or case-control studies; and 3) publications were restricted to English (from 2000 to 2022). An exposure assessment framework was made to determine whether a study was sufficiently TRAP-specific, namely the selection of traffic-related air pollutants, the exposure assessment method, and the spatial resolution. For example, we excluded PM studies where the exposure assessment was solely derived from monitoring data or studies that directly used averaged air pollution concentrations in the specific district as an individual exposure level [26, 28].

### Data extraction

Two reviewers (Lifang Liu and Jingxuan Ma) independently extracted the parameters of each study included in this systematic review, first author, year of publication, country of origin of the study, study design, the age of the participants, sample size, exposure assessment methods, the adjusted effect size, the corresponding 95% CI and adjustment variables. Disagreement was resolved by discussing, examining, and negotiating with a third reviewer (Shanshan Peng).

### Assessment of risk of bias in the included studies

As no randomized controlled trials were included in this systematic review, we used the Newcastle Ottawa quality assessment scale (NOS) to assess the quality of cohort and case-control studies. In this study, if the NOS score greater or equal to 7 were grouped as “high quality”; otherwise, the study was grouped as “low quality”.

## Results

**Fig 1:**
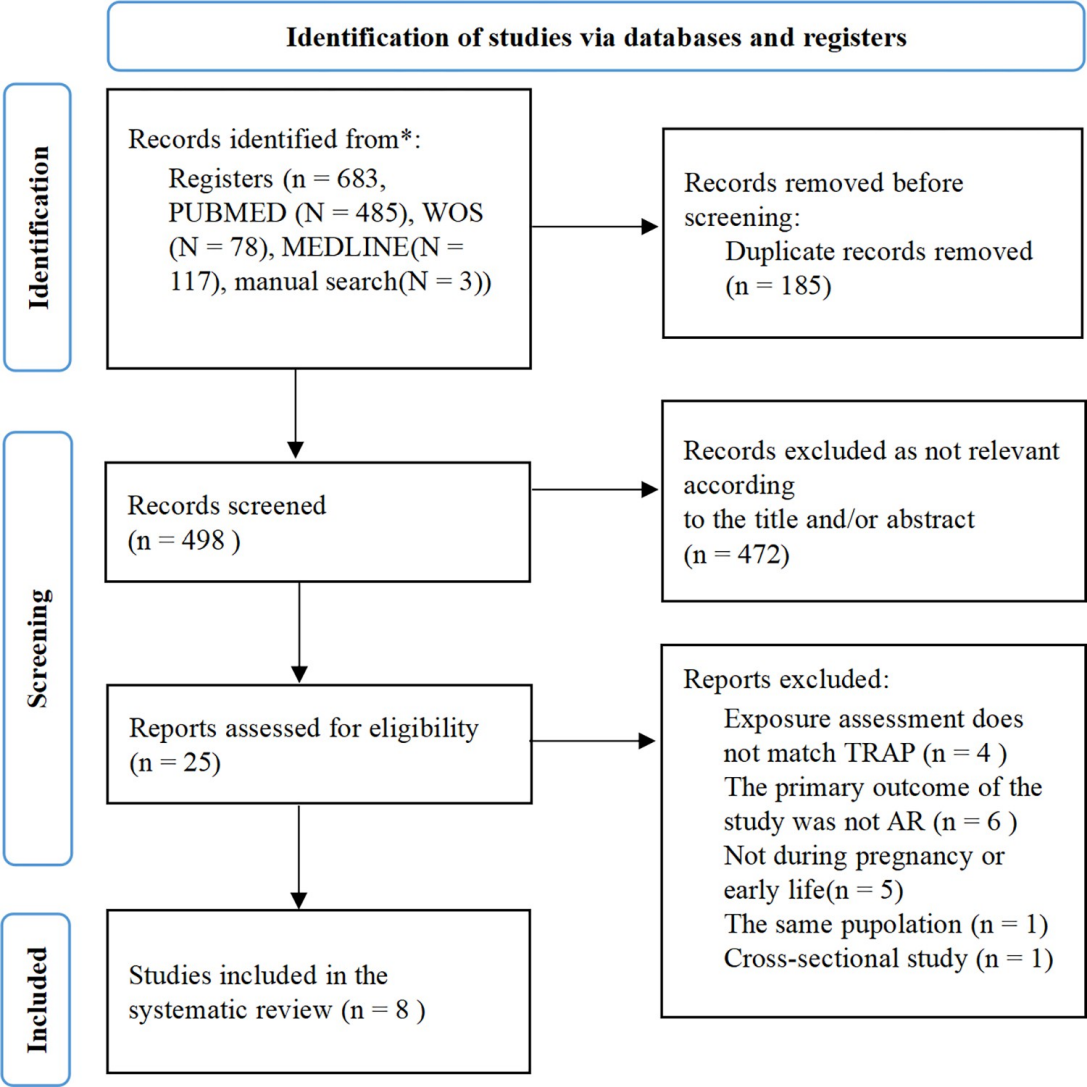
Flow chart of the study selection.

Fig 1 shows the study selection flow chart. A total of 498 relevant articles were retrieved through the pre-developed search strategy and reference search. After screening the titles and abstracts and a subsequent full-text review, eight studies were included in the systematic review.

The main characteristics and results of the included studies were shown in the Table 1. For each study, we extracted quantitative results from the single-pollutant model and adjusted OR/HR. Six of them were cohort studies and two were case-control studies. Six studies were conducted in China covering 9 cities located in the east, west, north and south regions, one in Canada and the other in Germany. All studies investigated the relationship between air pollution and AR, with three studies investigating during pregnancy and early life, two studies investigating during pregnancy, and three studies investigating early life. All were published within the past 6 years except for one published in 2008. The specific air pollutants evaluated in studies included PM_2.5_, PM_10_, NO_2_, BC, CO, and O_3_. Seven studies used validated models or weighted methods to assess air pollution exposures of individuals, and one used air monitoring station concentrations as personal exposure, these monitoring stations are located approximately 1000 m from each participant’s home and are mainly located in major traffic near the roads. Since the exposure assessment indicators and study types were heterogeneous and the fewer number of studies included in the review, a quantitative synthesis of the data was not possible.

**Table 1:** Characteristics and main results of the studies on the association between traffic-related air pollution and allergic rhinitis.

Author publication year [Ref]; setting	Study type;time period	Sample type,age and size	Outcome assessment	TRAP exposures and models used	Results	Adjustment variables
Tianyi Chen et al. [22] 2022;China	longitudinal study; 2011–2012	aged 3–6 years old; n = 23,934	ISAAC questionnaire, ICD-10	PM_2.5_, BC; Combined Geoscience-Statistical method	During pregnancy: IQR increase and prevalence ratio (PR) of DDAR^a^: 20.4 μg/m^3^ increase in PM_2.5_ (**PR = 1.43;1.11 to 1.84**), 1.0 μg/m^3^ increase in BC^b^ (**PR = 1.42;1.21 to 1.66**); IQR increase and prevalence ratio (PR) of **current hay fever**: 20.4 μg/m^3^ increase in PM_2.5_ (**PR = 1.84;1.35 to 12.5**), 1.0 μg/m^3^ increase in BC (**PR = 1.58;1.31 to 1.9**)	air pollutants, temperature, age, gender, family history of allergies, exclusive breastfeeding, family size, home dampness, home secondhand smoke exposure during the pregnancy, living area and household annual per capita income
YuTing Lin et al. [21] 2021;Taiwan, China	cohort study; 2005–2014	Infants; n = 140,911	ICD-9	PM_2.5_; Land-use Regression	IQR increase and incident AR:17.98 μg/m^3^ increase in PM_2.5_ (**HR = 1.27;1.17 to 1.37**) during pregnancy; (**HR = 1.60;1.46 to 1.74**) during infancy	maternal atopy, gender, socioeconomic status, maternal heart disease, maternal smoking and preterm birth
Yu Huang et al. [20] 2021;Taiwan, China	cohort study; 2000–2014	mother–newborn pairs; n = 982	Questionnaire	CO,O_3_,NO_2_, PM_10_; Weighted k- nearest neighbour	During pregnancy: High NO_2_, CO, and SO_2_ group compared with all mild levels group (**OR = 3.61;2.38 to 5.48)**	maternal atopy and parental education
Shuai Hao et al. [30] 2021;China	case-control study; 2017–2018	aged 2–4 years old; n = 388	Clinically diagnosed	PM_10_,NO_2_,CO; no model used	During 2 years of age to the day of AR diagnosis: IQR increase and prevalence of AR: 20μg/m^3^ increase in PM_10_ (**OR = 1.31;1.08 to 1.90**), 18μg/m^3^ increase in NO_2_ (**OR = 1.15; 1.02 to 2.23**), 354μg/m^3^ increase in CO (OR = 1.13;0.77 to 2.02)	sex, birth weight, delivery, feeding, eczema, parental allergy, stress, tobacco smoke, new furniture, pets and house redecoration
Teresa To et al. [29] 2020;Canada	cohort study; 2006–2016	aged 5–9 years old; n = 1286	ICD-9 and ICD-10	PM_2.5_, NO_2_; geographically weighted regression and land-use regression model	During the first 3 years of life: IQR increase and incident AR: 1.57 μg/m^3^ increase in PM_2.5_ (HR = 0.94;0.85 to 1.04), 4.16 ppb increase in NO_2_ (HR = 0.94;0.87 to 1.02)	age, sex, parental education level, income adequacy, number of people in household, low birthweight, breastfeeding, enrolment in childcare, whether child was born within 3 weeks of due date, home exposures during first year of life (damp spots, use of gas to cook or heat, exposure to environmental tobacco smoke, pets, cockroaches and mould) and parental history of asthma and atopy
Qing Huang et al. [2] 2019;China	case-control study;2017	aged 3–6 years old; n = 3165	ISAAC questionnaire	NO_2_,PM_2.5_,PM_10_; random forests model	1 μg/m^3^ increase and prevalence of AR: NO_2_ (OR = 0.979;0.972 to 0.987), PM_2.5_ (**OR = 1.232;1.201 to 1.263**), PM_10_ (**OR = 1.063;1.052 to 1.075**) during pregnancy; NO_2_(**OR = 1.013;1.002 to 1.025**), PM_2.5_ (**OR = 1.032;1.006 to 1.059**), PM_10_ (**OR = 1.018;1.005 to 1.031**) during the first year of life.	age, gender, maternal education, maternal occupation, paternal occupation, prepartal weight, gestational hypertension, birth season, eat milk powder, low birth weight, premature delivery, maternal secondhand smoke, paternal secondhand smoke, residential area
Qihong Deng et al. [8] 2016;China	cohort study; 2011.9–2012.1	aged 3–6 years old; n = 2598	ISAAC questionnaire	NO_2_,PM_10_;inverse distance weighted(IDW) method	IQR increase and prevalence of AR: 12 μg/m^3^ increase in NO_2_ (**OR = 1.41;1.05 to 1.88**), 12 μg/m^3^ increase in PM_10_ (OR = 0.98;0.75 to 1.28) during pregnancy; NO_2_ (**OR = 1.36;1.03 to 1.78**), PM_10_ (**OR = 1.54;1.07 to 2.21**) during the first year of life; NO_2_ (OR = 1.39;0.99 to 1.96), PM_10_ (0R = 1.27;0.92 to 1.75) after first year.	child’s sex, age, birth season, breast-feeding, and parental atopy, socioeconomic status and environmental tobacco smoke, new furniture, visible mold/damp stains, window condensation, cockroach and household dogs
Verena Morgenstern et al. [23] 2008; Germany	cohort study; 1999–2005	aged 4–6 years old; n = 2488	ISAAC questionnaire	PM_2.5_,PM_2.5_ absorbance,NO_2_; multiple linear regression model	During the first 6 years of life: IQR increase and prevalence of AR: 1.0 μg/m^3^ increase in PM_2.5_ (OR = 1.01;0.91 to 1.12), 0.2*10^– 5^m^−1^ increase in PM_2.5_ absorbance (**OR = 1.59;1.11 to 2.27**), 6.4 μg/m^3^ increase in NO_2_ (OR = 1.05;0.77 to 1.45)	sex, age, parental atopy, maternal education, siblings, environmental tobacco smoke at home, use of gas for cooking, home dampness, indoor molds, keeping of dogs and cats

^a^DDAR: Doctor-diagnosed allergic rhinitis

^b^BC: black carbon.

Nonetheless, in the studies on exposure during pregnancy, all studies found TRAP levels to be positively associated with the development of AR in children; in the studies on exposure during early-life, all studies found exposure to TRAP during the first year of life to be positively associated with the development of AR in children.

The results of the NOS are shown in Tables 2 and 3. Of the eight studies included, seven achieved 7 scores and above and one study achieved 6 scores.

**Table 2:** Newcastle–Ottawa scale to evaluate the methodological quality of cohort studies included in the systematic review.

Study	Year	Selection				Comparability		Outcome			Scale
		Exposed	Non- exposed	Ascertainment of exposure	Start without outcome present	Major factor	Addition factor	Outcome assessment	Follow-up length	Adequacy of follow up	
Tianyi Chen [22]	2022	1	1	1	1	1	1	1	0	1	8
YuTing Lin [21]	2021	1	1	1	1	1	1	1	1	1	9
Yu Huang [20]	2021	1	1	1	1	0	1	0	1	1	7
Teresa To [29]	2020	1	1	1	1	1	1	1	1	1	9
Qihong Deng [8]	2016	1	1	1	1	1	1	0	0	1	7
Verena Mo [23]	2008	1	1	1	1	1	1	0	0	1	7

**Table 3:** Newcastle–Ottawa scale to evaluate the methodological quality of case-control studies included in the systematic review.

Study	Year	Selection				Comparability		Exposure			Scale
		Adequate definition of cases	Representative-ness of cases	Selection of Control	Definition of control	important factor	Addition factor	Exposure assessment	Same method of ascertainment for cases and controls	Non-Response rate	
Shuai Hao [30]	2021	1	1	1	1	1	1	1	1	0	8
Qing Huang [2]	2019	0	0	1	0	1	1	1	1	1	6

## Discussion

To our knowledge, this systematic review is the first study to examine the impact of TRAP during pregnancy and early-life on AR in children. Seven of eight studies identified in the analysis were published within the last 6 years, that helped to provide support for linking TRAP (PM_2.5_, PM_10_, NO_2_ and CO) to AR.

Verena Morgenstern et al. [23] published the first study showing a relationship between PM_2.5_ absorbance during early life and the development of AR in children. In two prospective cohort studies, Qi-Hong Deng et al. [8] and Yu-Ting Lin et al. [21] further divided prenatal period into three trimesters (early pregnancy, mid-pregnancy, late pregnancy) and came to the consistent conclusion that the vulnerable time window may be late pregnancy and the first year of life for AR in children. Qing Huang et al. [2] conducted a case-control study of 3,165 preschool children from Wuhan and Ezhou, the results of this study highlighted that the effects of exposure to air pollution during pregnancy are stronger than exposure early in life. But the cumulative effect of the exposure period and the second year of life exposure to air pollutants were not significant with childhood AR. After a mean 17-year follow-up of 1286 children based on the Toronto Child Health Assessment Questionnaire (T-CHEQ) study, Teresa To et al. [29] found that early life exposure to ozone and nitrogen dioxide contributed to the development of asthma and eczema but no significant association with developing AR. Shuai Hao et al. [30] used the average daily concentration of pollutants from 2 years old to the day of AR diagnosis as the early childhood exposure level and considered the effect of floor level of residence, distance between home and kindergarten on exposure estimates. This case-control study found that preschool children exposed to PM_10_ and NO_2_ had an increased risk of AR by 70% and 85% respectively with family stress and male gender may increase the susceptibility to AR. In a 14-year follow-up birth cohort study, Yu Huang et al. [20] applied the latent class analysis to identify potential exposure patterns to air pollutants during pregnancy and concluded that high NO_2_, CO, and SO_2_ class had increased odds of AR development. Tianyi Chen et al. [22] used a combined longitudinal prospective study of 23 934 participants in 6 cities from 3 regions of China to measure the associations between exposure to air pollution and the development of AR in children. They further explored the effect of PM_2.5_ chemical composition on AR and found that maternal exposure to PM_2.5_ and chemical constituents, in particular BC, increased the risks of AR in preschool children.

Some of the mechanisms underlying the role of air pollution in the development of AR in children have been published in previous studies. Exposure to air pollutants during pregnancy could be transmitted to the uterus via the placenta. Recent studies have found evidence of the presence of black carbon particles in cord blood, confirming that these particles are able to cross the placenta and enter the fetal circulation system [31]. The development of human nasal mucosa starts at 8 weeks of gestation, by the third trimester, functional cells of the fetal nasal mucosa are basically formed, but the maturation of submucosal goblet cells and glands occur after birth [32]. Therefore, the period around birth is a critical stage in the development of the middle nose. Exposure to air pollutants during this period may destabilize the epithelial barrier function of the sinuses, leading to the development of allergic rhinitis and other immune disorders later in life. Prenatal and postnatal exposure to air pollutants is associated with early-life immune perturbations and affects the development of the nervous and neuroendocrine systems [33, 34]. Various epigenetic regulatory mechanisms linking early life exposure with subsequent development of AR are also gradually revealed [35].

The strengths of these studies are their overall high study quality, adequate sample size, reliable measurement of TRAP despite different methodologies, and a larger number of covariates controlled. The studies analyzed have some limitations. Firstly, exposure assessments of these studies were based on concentrations of air pollution estimated for the place of residence and the presence of subjects who changed their address during the study period. Therefore, misclassification of exposures cannot be completely avoided. Secondly, some studies used the ISAAC questionnaire or self-reports to determine the outcome, which lack confirmation of the disease diagnosis. Furthermore, the evaluation of exposure to contaminants was perhaps somewhat inaccurate, because several different exposure assessment methods that vary in their predictive power were used in these studies. Thirdly, although many potential confounders were adjusted in studies, residual confounding related to unmeasured confounders may still exist. Finally, pollutants of concern vary across studies, which made a systematic review difficult. In conclusion, this systematic review provides supportive evidence that exposure to TRAP during pregnancy and early-life increases the risk of developing AR in children.

## Suggestion for future directions

More studies are warranted to investigate a more exact association of TRAP, genetic factors as well as gene-environment interactions and allergic diseases including AR. Early life exposures are critical for postnatal respiratory development. In the future, it is important to elucidate the relationship between TRAP and childhood AR through large birth cohort studies, to clarify the time window of vulnerability, and to further explore gender and regional differences in the association between TRAP and AR and possible protective factors. The newly released WHO Global Air Quality Guidelines set new standards for air quality levels of six pollutants, further lowering the annual average target for PM_2.5_, PM_10_, NO_2_, SO_2_, and CO. These findings emphasized that regulatory authorities/government need more comprehensive air control policies to protect sensitive children, as well as raising public awareness of TRAP, and alert the public to the risk of AR.

## Supporting information

S1 Checklist PRISMA 2020 checklist. (DOCX)

S1 File (PDF)
