# Supplementary material for: Prenatal and early-life exposure to traffic-related air pollution and allergic rhinitis in children: A systematic literature review
Source: PLoS One. 2023 Apr 20;18(4):e0284625. doi: 10.1371/journal.pone.0284625 (PMC10118164; doi:10.1371/journal.pone.0284625)
Supplement: S1 File — (PDF) [file pone.0284625.s002.pdf]

NIHR National Institute for Health Research

PROSPERO
International prospective register of systematic reviews

Prenatal and Early-Life Exposure to traffic-related air pollution and allergic rhinitis in children: a systematic literature review

liu lifang, Yu wenao, Peng shanshan

To enable PROSPERO to focus on COVID-19 submissions, this registration record has undergone basic automated checks for eligibility and is published exactly as submitted. PROSPERO has never provided peer review, and usual checking by the PROSPERO team does not endorse content. Therefore, automatically published records should be treated as any other PROSPERO registration. Further detail is provided **here**.

Citation

liu lifang, Yu wenao, Peng shanshan. Prenatal and Early-Life Exposure to traffic-related air pollution and allergic rhinitis in children: a systematic literature review. PROSPERO 2022 CRD42022361179 Available from: https://www.crd.york.ac.uk/prospero/display_record.php?ID=CRD42022361179

Review question

Is there a relationship between Prenatal and Early-Life Exposure to traffic-related air pollutants and allergic rhinitis in children?

Searches

Studies were searched using the following electronic databases: PubMed, Web of Science and MEDLINE. The terms used in the search followed the rules of Medical Subject Headings (MeSH) and the question that the search was guided by the recommended PECOS strategy (participate, exposure, comparison, outcome and study).
Summary of our search terms: #1. Related to Pregnancy or Early Life: (Descriptors: "child health" or "childhood" or "adolescents" or "teenagers" or "children" or "Pre-School Children" or "Prenatal Exposure*" or "Early Life Exposure*" or "Pregnancy" or "Preconceptional exposure*" or "pregnancy exposure*"); #2 Related to Traffic-related air pollution: (Descriptors: "PM2.5" or "NOx" or "NO2" or "traffic related air pollution" or "traffic pollutant*" or "vehicle emission" or "nitrogen oxides" or "particulate matter" or "traffic exposure" or "automobile emission" or "traffic emission" or "proximity to roadways" or "proximity to major roads" or "ambient air pollution"); #3 Related to allergic rhinitis: (Descriptors: "allergic rhinitis" or "Rhinitis, Allergic")
We will use the following combination to search in the databases: #1 AND #2 AND #3.
We will not restrict the search by starting date (database inception until the day of the search). The email alert feature in PubMed will be used to avoid missing any study published after the final date of searches.

Types of study to be included

Prospective or retrospective longitudinal studies. Cohort studies.case-control studys.

Condition or domain being studied

allergic rhinitis.

Participants/population

To be eligible for inclusion, a published manuscript must report on a study sample drawn from Pregnancy population or children up to 12 years of age.

Intervention(s), exposure(s)

To be eligible for inclusion, a published manuscript must report on associations which measured/modeled ambient air pollution, using a minimum 3-mouths averaging period. These pollutants arise from motorised traffic . For air pollution regulatory monitoring data, The monitoring sites located in residential areas with no pollution sources nearby should be stated.Manuscripts not to mention traffic related air pollution or vehicle emission or living near motorways or distance to major roads woule be exclude. Inclusion criteria also require that the main effect of air pollution on allergic rhinitis, as described below, be a primary analysis of interest. If "main effects" of an air pollutant on the outcome have been reported in a given study population, we will exclude manuscripts that report results in this same cohort. Example: we will exclude articles focusing on effect modification (e.g., modification of air pollution's association with allergic rhinitis by genetic variant).

Comparator(s)/control

Not relevant.

Context

Estimating the main effect of ambient outdoor air pollution on allergic rhinitis in children must be the primary purpose of the study.

Main outcome(s)

To be eligible for inclusion, each published manuscript must report on associations with clinically allergic rhinitis or use the International Study of Asthma and Allergies in Children (ISAAC) questionnaire as a a standardized tool.
The definitions of doctor diagnosed diseases were referred to the International Classification of Diseases ICD-10, WHO 2016- AR (J30.401) .

Additional outcome(s)

None

Data extraction (selection and coding)

All identified articles will be downloaded into the EndNote X9 Reference Manager software, where they will be managed and stored. Duplicate papers will be removed. We will develop a data collection form for study characteristics, exposure assessment and outcome data. Two reviewers will extract study characteristics from included studies; any disagreements will be discussed in consultation with a third reviewer. We will extract the following study characteristics:
1. Study characteristics: Authors, year, country, number of study centres and location/s
2. Methods: study design, sample size, total duration of study, assessment points
3. Participants: population, gender, mean age, age range, socioeconomic status
4. Exposure: pollutants included and type/s of exposure assessment，concentration/level of pollutant

5. Outcome: outcome definition/s; effect estimates and 95% CI; how these estimates were derived
6. Adjustment for key confounders
7. Notes: notable conflicts of interest

Risk of bias (quality) assessment

Two review authors will independently assess quality of reporting for each study using the Newcastle Ottawa Scale for cohort and case-control studies. All articles will be classified into high, medium or low quality or unsatisfactory based on the quality score. Where multiple outcomes are assessed, each outcome will be assessed separately. If multiple studies report on the same cohort, each outcome will be assessed, and results merged. If multiple papers report on the same outcome in the same cohort, the papers with the larger number of participants or most recently published will take precedent. Full scores will be reported.We will resolve any disagreements by discussion or by involving a third review author. We may exclude any unsatisfactory papers if the methods and results are not sound and do not support the conclusion. We will assess common bias across the articles using the GRADE guidelines. We may perform funnel plots to address potential publication bias.

Strategy for data synthesis

Studies will have data extracted and synthesised into a narrative review, with data extraction table reported, including quality score and extracted fields as appropriate. Reporting within the review will be guided by the PRISMA statement with appropriate checklist and flowchart included.

Analysis of subgroups or subsets

Non applicable.

Contact details for further information

liu lifang
liulf9666@163.com

Organisational affiliation of the review

Sichuan University
http://wcsph.scu.edu.cn/

Review team members and their organisational affiliations

Ms liu lifang. Sichuan University
Mr Yu wenao. Sichuan University
Ms Peng shanshan. Sichuan University

Type and method of review

Epidemiologic, Systematic review

Anticipated or actual start date

08 September 2022

Anticipated completion date

30 December 2022

Funding sources/sponsors

Not applicable.

Conflicts of interest

Language

English

Country

China

Stage of review

Review Ongoing

Subject index terms status

Subject indexing assigned by CRD

Subject index terms

Air Pollutants; Air Pollution; Child; Female; Humans; Pregnancy; Rhinitis, Allergic

Date of registration in PROSPERO

29 September 2022

Date of first submission

19 September 2022

Stage of review at time of this submission

Stage	Started	Completed
Preliminary searches	Yes	No
Piloting of the study selection process	Yes	No
Formal screening of search results against eligibility criteria	Yes	No
Data extraction	No	No
Risk of bias (quality) assessment	No	No
Data analysis	No	No

The record owner confirms that the information they have supplied for this submission is accurate and complete and they understand that deliberate provision of inaccurate information or omission of data may be construed as scientific misconduct.

The record owner confirms that they will update the status of the review when it is completed and will add publication details in due course.

Versions

29 September 2022
29 September 2022

PROSPERO
This information has been provided by the named contact for this review. CRD has accepted this information in good faith and registered the review in PROSPERO. The registrant confirms that the

information supplied for this submission is accurate and complete. CRD bears no responsibility or liability for the content of this registration record, any associated files or external websites.
